# The retirement of Editor-in-Chief Arne Andersson, Upsala Journal of Medical Sciences 2006–2022: an amazing journey under Arne’s stewardship

**DOI:** 10.48101/ujms.v127.8630

**Published:** 2022-03-14

**Authors:** Gerhard Wikström, Michael Welsh

**Affiliations:** Institute of Medical Sciences, Uppsala University Hospital, Uppsala, Sweden; Department of Medical Cell Biology, Uppsala University, Uppsala, Sweden

The day we have dreaded for several years has finally come. Our Editor-in-Chief (EIC), Professor Arne Andersson, has decided to retire after serving that role since 2006. During those years, Upsala Journal of Medical Sciences (UJMS) experienced a remarkable transformation from a local journal with an impact factor of 0.476 to a global open access player with a 2-year impact factor of 2.384 (5-year impact factor is 3.080). Ever since it was founded in 1865, UJMS has been our precious ‘little gem’ (1, 2). The journal is under the auspices of the Uppsala Medical Society (Upsala Läkareförening) and has served an important role as a venue for scientific exchange within the local medical community. The journal’s recent development has allowed Uppsala medical science to thrive in an increasingly global environment, and the increased relevance of our Journal is the consequence of Arne Andersson’s focused stewardship.

Arne Andersson is a distinguished diabetes researcher who made important contributions with particular focus on pancreatic islet transplantation. He shared the role as EIC with Gunnar Ronquist from 2006 to 2012 after which he became a sole EIC. He grasped at an early stage the ongoing evolution of medical science with its diversification and specialization, and consequently, he implemented changes in journal policies to address this. This resulted in the establishment of an editorial team of associate editors and an editorial board including international members. He accepted 493 manuscripts as EIC after scrutinous review, always with the highest’s scientific standards. His personal commitment to this process includes supervision of linguistic text revision in order for manuscripts to reach impeccable standards. The extreme diversity of high-quality publications recently accepted can be illustrated by publications in the fields of antibiotic resistance (3), vascular biology (4), diabetes (5–7), microRNA in tumor biology (8, 9), fertility (10), point-of-care testing (11), and clinical aspects on phage therapy against bacterial infections (12).

The journal has experienced additional changes during Arne Andersson’s leadership. It has become an open access online journal, initially with both electronic and printed paper versions of all articles but since 2021 only with electronic publications. That transition was a necessity due to conditions imposed by the COVID-19 pandemic, and Arne Andersson had mixed feelings about this change, always preferring the printed version for reading.

We are immensely grateful to Arne Andersson for his sublime input to our Journal, and our intentions are to maintain the course set out by him.


Gerhard Wikström Institute of Medical Sciences, Uppsala University Hospital, Uppsala, Sweden
Michael Welsh Department of Medical Cell Biology, Uppsala University, Uppsala, Sweden

